# Acupuncture Points and Perforating Cutaneous Vessels Identified Using Infrared Thermography: A Cross-Sectional Pilot Study

**DOI:** 10.1155/2019/7126439

**Published:** 2019-03-21

**Authors:** D. Álvarez-Prats, O. Carvajal-Fernández, F. Valera Garrido, D. Pecos-Martín, A. García-Godino, M. M. Santafe, F. Medina-Mirapeix

**Affiliations:** ^1^Clínica Fisioterapia Océano, Servicio de Fisioterapia, Calle las Palmas, 55, 28938 Móstoles, Madrid, Spain; ^2^Servicio de Fisioterapia MVClinic, Pozuelo de Alarcón, Madrid, Spain; ^3^Pain and Physiotherapy Group, Department of Physiotherapy, University of Alcalá, Madrid, Spain; ^4^Clínica Fisiopuntura, Servicio de Acupuntura y Fisioterapia, Calle Alonso Heredia 7, 28028, Madrid, Spain; ^5^Unit of Histology and Neurobiology, Department of Basic Medical Sciences, Faculty of Medicine and Health Sciences, Rovira i Virgili University, Carrer St. Llorenc, No. 21, 43201 Reus, Spain; ^6^Department of Physical Therapy, Regional Campus of International Excellence “Campus Mare Nostrum”, University of Murcia, Murcia, Spain

## Abstract

**Aims:**

To evaluate the presence of perforating cutaneous vessels (PCV) in different lower limb acupuncture points (AP) using thermography.

**Material and Methods:**

An analytical cross-sectional study was performed on the two lower limbs (n=6) of volunteer subjects. In total, 144 AP and 144 control points (CP) were analysed, one for each AP. First, the AP and CP were located on each individual. Subsequently, both the real and thermographic images were created. In the real images, the location of the AP and the established CP were highlighted with boxes. FLIR Tools Plus and Physio Thermal Imaging software were used to merge the real image with the AP and the CP and to merge the thermographic image with the PCV. By superimposing both images, we were able to verify the presence of PCV among the AP and CP.

**Results:**

PCV were identified in 87.5% of the 144 AP examined and in 18.1% of the respective CP. All the AP had a higher percentage of PCV compared to their respective CP, with statistically significant differences in all points, except for ST33 and ST34. The probability of finding PCV in AP was 11 times higher than the probability of not finding it.

**Discussion:**

Thermography may serve as a useful tool in the assessment and treatment of patients using acupuncture. The presence of PCV in the area of the acupuncture needle insertion could partially influence the effects generated by the acupuncture technique from the vascular autonomic point of view.

**Conclusions:**

There is a high proportion of PCV in the AP area located in the lower limb.

## 1. Introduction

Acupuncture is an invasive technique used for diagnosis and treatment and is a part of the Traditional Chinese Medicine. This technique involves inserting fine metal needles upon points located in predefined areas known as acupuncture points (AP), which belong to a network of parallel and symmetric lines running along the trunk and the limbs, which are known as meridians [1].

The use of acupuncture has become widespread and, today, is commonplace in the so-called Western medicine, to such extent that, over the last ten years, over 14,000 articles have been published on acupuncture and AP in the scientific community (according to a PubMed search using the keywords “Acupuncture” AND “Acupoints”, August 2017).

Many authors have researched exactly what is stimulated when a needle is inserted into an acupuncture point, and even whether acupuncture points do actually exist [2]. In recent years, a number of studies have been conducted in an attempt to research the connection between these points and different structures with the aim of understanding exactly how acupuncture works. Anatomical relationships have been suggested, such as the overlapping of AP with interfascial planes [3], myofascial trigger points [4], peripheral nerves [5], and blood vessels [6].

The relationship between the acupuncture points and the vascular system is stated in the Yellow Emperor's Canon of Medicine (Huang Di Nei Jing). Following the description made in one of the passages of this book regarding the Chong meridian, which stated that “when the pathological energy of the cold invades the* Chong* mai, it leads to the stagnation of the blood of this extraordinary canal...”, Vivien Shaw performed a cadaveric dissection and, thereafter, concluded that this coincided precisely with the description of the main vascular pathways [7]. In 1988, Hartmut Heine performed a cadaveric study in which he identified the AP as perforations of the* corporis superficialis* fascia, which comprised two veins and a small artery, accompanied by a nerve [8]. These neurovascular structures discovered by Heine are similar to the anatomic description of perforating cutaneous vessels (PCV).

Perforating cutaneous vessels are vascular structures which penetrate the most external layer of the deep fascia in order to provide vascular flow to the subcutaneous cellular tissue, and which have been widely studied in the field of cosmetic and reconstructive surgery for the design of grafts [9]. Different methods are available for locating PCV, such as hand held Doppler, Doppler colour ultrasound, computed tomography angiography, magnetic resonance angiography [10], dynamic infrared imaging with no thermal challenge [11], infrared thermography after cooling [12], and infrared thermography after the use of tourniquet [13].

Infrared thermography is a complementary diagnostic tool which enables the real-time visualization and assessment of the skin temperature and which generates a thermal map of the skin surface [1]. The human body, in order to maintain its internal thermal homeostasis, exchanges heat with the environment via conduction, convection, evaporation, and infrared radiation. The energy emitted in the form of infrared radiation represents 60% of the total [14], of which, 80-90% does so with a wavelength of 8-15*μ*m [1]. Thermal cameras are able to capture the infrared radiation emitted by the skin surface in this wavelength and, thereafter, generate a thermal map of the human body.

In turn, the skin temperature is a reflection of the amount of skin blood flow [15]. For this reason, thermography enables us to precisely determine the location of the PCV, if an appropriate provocation stimulus (cold, compression, postural,…) was performed in the form of the so-called ‘hot spots' [11–13].

The aim of the present study was to use infrared thermography as a descriptive tool to provide support for the use of acupuncture and to establish a possible association between AP and PCV. To increase the external validity of our results, we have examined and compared the frequency of PCV using a total of 12 AP with nonacupuncture points, or control points (CP), located on the same meridian and used as their respective control. Our hypothesis was that the frequency of PCV identified using thermography would be greater in the AP studied than in their respective control points.

## 2. Material and Methods

### 2.1. Design and Study Units

**Figure 1:**
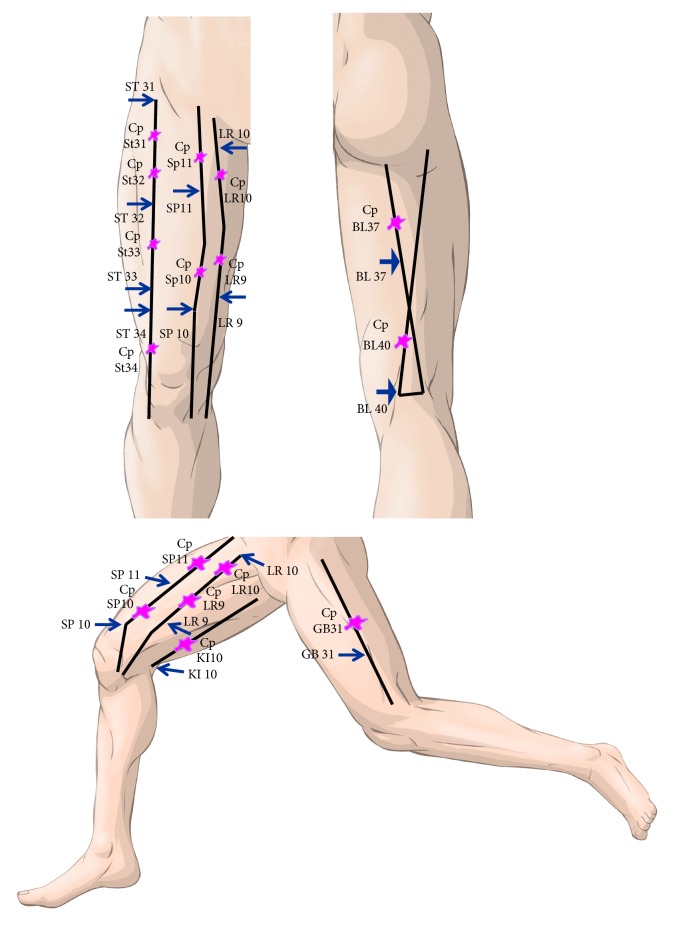
Modified from Focks C. Atlas of acupuncture, China, Elsevier Churchill Livingstone, 2006. The image shows the classic description of the meridians of the lower limb used in this study. The arrows indicate the acupuncture points while the symbols shown in purple are the CP, one for each AP along the same meridian.

A cross-sectional analytical study was performed, in which the presence of PCV was thermographically identified on a series of 12 AP and 12 CP previously identified on both sides of the body by physiotherapists who had experience in the field of infrared thermography and invasive physiotherapy. For each AP, its respective CP was located systematically within the same meridian of the AP at four centimetres cranial or caudal to the same. As such, the area selected as the CP did not contain any acupuncture points (Figure 1). One of the physiotherapists was in charge of locating the different AP and CP, whereas the other was in charge of recording and processing the infrared images. The 12 AP were as follows: points located on the anterior aspect of the thigh (stomach meridian 31, 32, 33, and 34), the lateral aspect of the thigh (meridian of the gallbladder 31), the posterior aspect (meridian of the bladder 37 and 40, meridian of the kidney 10), and the medial aspect (liver meridian 9 and 10, spleen meridian 10 and 11) (Table 1). The Ethical Research Committee (ERC) of the CEU San Pablo University approved this study, which followed all the principles established in the Helsinki Declaration.

The study participants comprised six subjects (50% male and female) with a mean age of 36 years (range 28-40) who were recruited in April 2015 among patients of the Fisiocéano clinic (Móstoles, Madrid, Spain). The exclusion criteria were the presence of systemic pathologies and/or the consumption of substances and drugs that could alter the normal physiological behaviour of the nervous system and, therefore, provoke alterations in the cutaneous blood flow. All subjects signed the corresponding informed consent in order to participate in the study.

### 2.2. Procedure

Firstly, for each subject, all 24 AP and CP were located on both sides of the body via the ruler method. Ultrasound was also used to support the location of 3 AP (bladder 40, kidney 10, and stomach 31), as they were located on the anatomic landmark areas (Table 1).

The ruler method consists of assigning the Chinese measurement* ‘cun' *(the width of the interphalangeal joint of the first finger) which is traditionally the measurement described in numerous acupuncture manuals and atlases[16, 17], with a specific individual measurement in centimetres. This method has been validated by different authors, such as Aird M. et al., who have defined it as being the most precise for the interexaminer location of points [18]. Using this method, the precision of AP location is 2.90 cm^2^. This area has been used in the present study as a reference area for the AP and CP in order to validate the presence of PCV in the same way.

In order to calculate the value of a* “cun” *in centimetres, an imaginary line was measured between the pubic symphysis and the medial femorotibial interline, the equivalence of which, according to the different atlases, is 20 cun [16, 17]. This measurement was performed three times on both legs of each individual, and subsequently the mean of the distance obtained for each leg was calculated. Finally, the mean of the result in centimetres was divided into 20 cun, to obtain the specific* “cun”* value in centimetres for each person and for each leg.

The location of the AP and CP was performed while standing and the site area was marked using a skin marking pen (Comed) and a 2.90 cm^2^ template. Once all AP and CP, placed at 4 cm from the centre of the area of the AP, were marked, the location of PCV was achieved via the thermographic study. We have used this measure based on the results found by Aird M. et al. According to this study [18], the proportional method is the most recommended measurement system because it prevents inserting a needle into an incorrect location based on the Traditional Chinese Medicine concept. The maximum radius measurement of the ellipse generated by the proportional method never exceeded 4 cm. Therefore, we have considered this measure to establish the CP.

For this purpose, all subjects were subjected to a 15-minute acclimatisation period by standing with bare legs in a room at 22-24°C, thus fulfilling the criteria for the performance of thermographic studies according to the guidelines established by the American College of Clinical Thermography [14].

Under normal conditions, PCV go unnoticed in the thermal image of a patient who is correctly acclimatised, and the subject must undergo a process of vascular stimulation in order to make this evident. Some studies have used thermal or compression stress to achieve this objective [12, 13]. In the case of thermal stress, cooling of the subject's skin was done to produce a vasoconstriction and, subsequently, taking thermal images during the process of skin reheating occurs via the PCV [12, 19]. In this study, subjects were placed with their legs raised above the heart level. This posture provokes a skin vasodilation which occurs as a consequence of the activation of the venoarteriolar skin reflex [20]. To achieve this, patients were placed lying with their legs bare on the treatment table (having been previously acclimatised), with their legs raised above heart level over a period of five minutes. Afterwards, the subjects stood up and six thermographic images of the legs were taken in order to capture all the AP perpendicularly. Thus, optimising the infrared radiation emitted by the area [21].

Images were captured using a thermographic FLIR E60 camera (resolution 320 x 240; FOL18 lens; thermal sensitivity of 0.05°C (50mK); serial number 64509645099).

**Figure 2:**
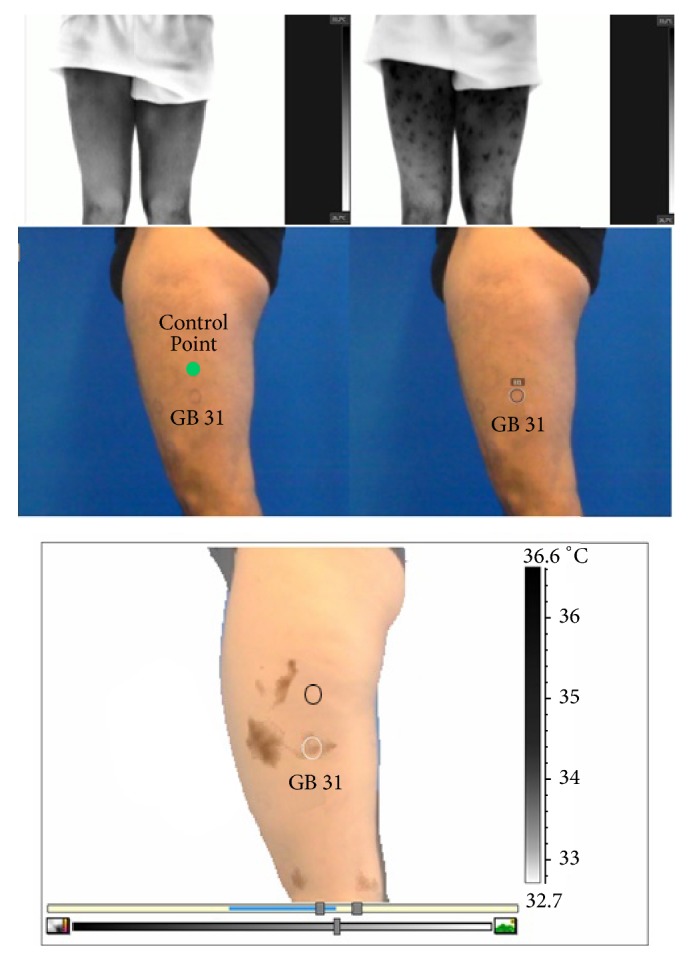
The image shows the different phases of the technical process of capturing the thermogram as well as the different types of computer processing. The thermal image in the acclimatisation period (upper left); image after provoking the venoarteriolar skin reflex (upper right); real image showing AP markings (middle); thermal merging (lower image).

Subsequently, the real images taken were processed using* Physio Thermal Imaging software* and* FLIR Tools Plus software* (*Quick Report*). In the first place, the real image was marked by drawing circular boxes on the skin surface of the area where the AP and PC were located. Then, the thermal image was optimised (adjusting the range, level, and colour palette to highlight the PCV that appeared in the same way (Figure 2)). Lastly, the MSX function of the software was used, enabling the merging of images (real and thermal), improving of the contrast of the image, and recording of the data regarding overlaps, “yes or no” of the previously marked AP and the CP, and the presence of a PCV in the area was determined using a qualitative analysis for thermal differences (Figure 2).

### 2.3. Statistical Analysis

Data on the frequency of the PCV in each of the 12 AP and their respective CP, as well as the ensemble of these, were summarised via proportions (%) and odds, with their respective 95% confidence interval (95% CI). The odds were calculated using the following formula: odds=proportion / 1-proportion.

To analyse the association between the AP and PCV, the ratio of proportions (RP) and odds ratio (OR) of the AP and their CP (with their respective 95% CI) were calculated singly and in combination. The formulas used were: RP= proportion in the AP /proportion in the CP; OR= odds in the AP /odds ratio in the CP. A significant association was established when the 95% CI of the ratios included 1. All the analyses were performed using the SPSS program, version 21.0.

## 3. Results

**Figure 3:**
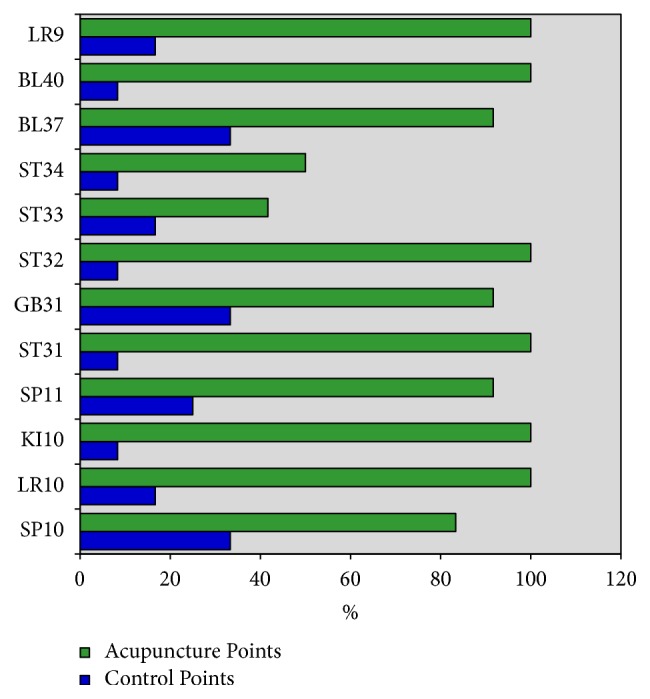
The image shows the percentage of acupuncture and control points with PCV.

In general, PCV were identified in 87.5% of the 144 AP examined (24 AP/subject; 12 AP in each lower limb) and in 18.1% of the respective CP (Table 2). The AP that displayed PCV in all cases were BL40, KI10, ST31, ST32, LR9, and LR10. In contrast, the AP with a lower frequency of PCV were ST33 and ST34. Nonetheless, all the AP had a greater percentage of PCV than their respective CP (Figure 3). When we compare AP and CP in terms of proportions, the differences are close to what is expected. In all points, the ratio was greater than 1. In other words, there was a greater probability of finding PCV in the AP than in the CP, with statistically significant differences in all points, except two (ST33 and ST34). The greatest ratio value was 12 (IC 95%: 1.8-78.4) and this was obtained in the following points: BL40, KI10, ST31, and ST32.

In the BL40, KI10, ST31, ST32, LR9, and LR10 AP, it was not possible to calculate the odds for the presence of PCV because the denominator of the ratio was 0%. Therefore, the points with a greater calculated odds ratio were BL37, SP11, and GB31. At these points, the probability of finding PCV was 11 times greater than the probability of not finding one. The odds of PCV in the CP were less than one in all cases. In other words, the probability of not finding a PCV was greater in these points. When we compared AP and CP via the odds ratio, we observed significant ORs and ORs that are either equal and higher than 10 in all cases, except in the case of AP ST33.

## 4. Discussion

According to the results obtained, an elevated presence of PCV exists in the location of the AP studied, compared to the presence of PCV in CP, as well as a greater probability of finding PCV in an AP rather than in a CP where no acupuncture point is described. Of the AP analysed, only in AP ST33 and ST34 did we fail to find statistically significant results; both of which are points located on the anterior aspect of the thigh, close to the knee joint. These findings may be due to, in part, the lack of coincidence due to the anatomic variability among subjects or, possibly, because these vessels were not located with a processing by simple qualitative analysis due to thermal differences [13]. A study conducted by Muntean et al. demonstrated (with a 95% CI) that for the location of PCV, infrared thermography has a sensitivity of 95.05% and a precision of 77.41%, and its use for the location of PCV was optimised in combination with colour Doppler ultrasound [22]. Therefore, it would be recommendable to evaluate those points in which the presence of PCV is lower by using other processing imaging methods or systems to locate PCV [10]. This would be helpful in order to confirm whether a coincidence really exists, bearing in mind the clinical interest of these AP (ST33, ST34) for the application of clinical acupuncture in pathologies affecting the anterior aspect of the knee.

Each PCV consists of an artery and a vein which emerge from greater depths via a source vessel that normally runs parallel to the bone structures. Two types of PCV exist according to the path they follow until they get to the surface of the skin: the septocutaneous kind, which travels via the intermuscular septa (direct perforators) and the musculocutaneous kind, which travel through the muscle structures (indirect perforators). These two PCV perforate the most superficial layer of the deep fascia in order to irrigate the subcutaneous cellular tissue and the skin [9]. These findings confirm the results obtained by Harmunt Heine in his cadaveric studies and in which AP are described as structures comprising an artery, two veins, and a nerve which run through the* fascia corporis superficialis* [8].

Acupuncture points have been the subject of many studies over the years. Authors such as Langevin HM et al. or Bai Y et al. have demonstrated the relationship between the location of the AP and the interfascial planes [3]. Dorsher P et al. established a correlation between myofascial trigger points and AP [4]. On the other hand, some authors have related AP to richly vascularised [6] and innervated structures [5]. In the present study, the presence of PCV has been demonstrated in AP; this hypothesis has recently been published by Ding Zhi Wei [23], and this could be another explanation for the effects of acupuncture, as the insertion and mechanical and/or electrical stimulation of an acupuncture point in a richly vascularised and innervated area, such as a PCV, may activate mechanisms via both the peripheral sensitive nervous system and the sympathetic autonomous nervous system which may explain the local, segmental, and central effects that occur with this technique [24, 25].

On the other hand, one of the issues that many authors insisted on, representing the focus of many studies, is the exactitude regarding the location of AP. According to Hartmun Heine [8], the diameter of an AP ranges between 2mm and 8mm, and according to Sussmann D. [16], the size of an AP is 2mm^2^. However, the precision of the interexaminer location of an acupuncture point varies according to the method used for its location. According to a study performed by Aird M et al., in which 72 acupuncture practitioners participated with varying levels of experience, the location of an AP with a 95% CI for the same point in the same subject varied between an area of 12.7 cm^2^ with the most imprecise traditional method and an area of 2.9 cm^2^ using the ruler method employed in the present study [18]. Nonetheless, studies performed with CAT and thermography have confirmed that the effects of acupuncture depend on whether the needling is truly performed on a real AP [26]. This is due to the presence of specific cells and an increased density of receptors per mm^2^ in the AP compared to a “neutral” skin area [27, 28]. These apparently contradictory data suggest that there should be an area surrounding the AP which, when sufficiently stimulated, may produce the desired therapeutic effects. As can be observed, the richly vascularised and innervated area typical of the presence of a PCV, is greater than 2 mm^2^. This may explain why in some cases, despite the clinical inaccuracy of the insertion of the needle on the AP, the needling may still be effective.

Regarding thermography, this is a tool that is already being used in the therapeutic application of acupuncture for the diagnosis and selection of points and meridians to be stimulated, for a real-time assessment of the effects produced by the intervention, and for patient's reassessment. Wu ZY et al. have demonstrated the existence of a temperature gradient among the location of the AP and the surrounding tissue [29]. Dong Zhang performed a study comparing the selection of points for acupuncture treatment with thermographic assistance, compared to a control group of conventional acupuncture treatment in facial paralysis. The results revealed a healing rate in the thermography group of 90% compared to 77.5% in the control group, as well as a reduced number of sessions and a shorter treatment duration [30]. Furthermore, Shuy-yinlo conducted a study in which a diagnosis was performed according to the symptoms and the thermographic findings in the patient's area of pain. Based on this assessment, they selected a single point at a distance from the patient's area of pain for the needle insertion, while using thermal imaging to objectively observe how a decrease in temperature of between 0.5°C and 2°C occurred in the symptomatic area. This coincided with pain relief and did not occur in the patient's areas of pain that were not related to the selected meridian [31].

### 4.1. Study Strengths and Implications for Clinical Practice

This is the only study till date that integrates infrared thermography, the concept of PCV, and AP. One of the strengths of our study is the potential of opening new lines of research. Our findings are useful for clinicians, bearing in mind that the presence of PCV in the area of the acupuncture needle insertion may partially influence the effects generated by the acupuncture technique, from the vascular autonomic vascular point of view. Furthermore, these results suggest that infrared thermography may be a powerful tool for locating AP in the clinic. This has many implications for both the therapist and the patient: enabling the straightforward identification of target areas during needling, as well as providing patients with a powerful visual feedback.

## 5. Conclusions

There is an elevated presence of PCV in the AP selected for this study. Thermography could be a useful tool both for the location of AP as well as for the process of physiotherapy care in the application of acupuncture.

## Figures and Tables

**Table 1:** Description of the acupuncture points used in the study.

Point	Meridian	Assessment method
BL37	Urinary Bladder	8 *cun* vertical cranial direction from BL40

BL40	Urinary Bladder	Centre of the popliteal fossa (between the tendons of the biceps femoralis and the
		semitendinosus). *Ultrasound localisation.*

KI10	Kidney	Centre of the popliteal fossa (between the tendons of the semimembranosus and
		semitendinosus).* Ultrasound localisation.*

ST31	Stomach	Inferior to the AIIS, at the intersection with the sartorius. *Ultrasound localization.*

ST32	Stomach	11 *cun* caudal from ST31 or 6 cun cranial from external top of the patella, in the
		imaginary line that joins ST31 with the external portal of the knee (ST35)

ST33	Stomach	14 *cun* caudal from ST31 or 3 cun cranial from external top of the patella, in the
		imaginary line that joins ST31 with the external portal of the knee (ST35)

ST34	Stomach	15 *cun* caudal from ST31 or 2 cun cranial from external top of the patella, in the
		imaginary line that joins ST31 with the external portal of the knee (ST35)

SP10	Spleen	4 *cun* cranial (in a palpable depression of the vastus medialis)

SP11	Spleen	10 *cun* cranial (at the intersection between the sartorius and the vastus medialis)

GB31	Gall bladder	12 *cun* from the highest prominence of the greater trochanter in the caudal
		direction, on the midline of the outer thigh; when the patient is standing in the
		military position with hands close to sides, the point is at the tip of their middle
		finger

LR9	Liver	6 *cun* from the internal femorotibial joint line (intersection between the sartorius
		and the vastus medialis)

LR10	Liver	3 *cun* inferior to the superior border of the pubic symphysis (intersection
		between the Sartorius and the adductor longus)

**Table 2:** Differences in the frequency of the PCV among the different acupuncture and control points.

	Acupuncture points		Control points		Proportion ratio (95% CI)	Proportion Odds Ratio (95% CI)
	(n=12)		(n=12)			
	%	Odds	%	Odds		
	(95% CI)	(95% CI)	(95% CI)	(95% CI)		
BL37	91.7	11	33.3	0.5	2,8 (1.2-6.2)*∗*	22 (2.1-236)*∗*
	(64.4-98.5)	(1.8-65.7)	(13.8-60.9)	(0.2-1.6)		

BL40	100	unknown	8.3	0.09	12 (1.8-78.4)*∗*	unknown
	(75.8-100)		(1.5-35.4)	(0.01-0.5)		

KI10	100	unknown	8.3	0.09	12 (1.8-78.4)*∗*	unknown
	(75.8-100)		(1.5-35.4)	(0.01-0.5)		

ST31	100	unknown	8.3	0.09	12 (1.8-78.4)*∗*	unknown
	(75.8-100)		(1.5-35.4)	(0.01-0.5)		

ST32	100	unknown	8.3	0.09	12 (1.8-78.4)*∗*	unknown
	(75.8-100)		(1.5-35.4)	(0.01-0.5)		

ST33	41,7	0,7	16.7	0.2	2,5 (0.6-10.5)	3.6 (0.5-24)
	(19.3-68.1)	(0.2-2.1)	(4.7-44.8)	(0.1-0.8)		

ST34	50	1	8.3	0.09	6 (0.8-42.6)	11 (1.1-114.1)*∗*
	(25.4-74.6)	(0.3-3)	(1.5-35.4)	(0.01-0.5)		

SP10	83,3	5	33.3	0.5	2,5 (1.1-5.8)*∗*	10 (1.4-69.3)*∗*
	(55.2-95.3)	(1.2-19)	(13.8-60.9)	(0.2-1.6)		

SP11	91.7	11,5	25.0	0.3	3,7 (1.4-9.9)*∗*	33 (2.9-374.3)*∗*
	(64.4-98.5)	(1.8-65.7<)	(8.9-53.2)	(0.1-1.1)		

GP31	91,7	11,5	33.3	0.5	2,8 (1.2-6.2)*∗*	22 (2.1-236)*∗*
	(64.4-98.5)	(1.8-65.7<)	(13.8-60.9)	(0.2-1.6)		

LR9	100	unknown	16.7	0.2	6 (1.7-21.3)*∗*	Unknown
	(75.8-100)		(4.7-44.8)	(0.1-0.8)		

LR10	100	unknown	16.7	0.2	6 (1.7-21.3)*∗*	unknown
	(75.8-100)		(4.7-44.8)	(0.1-0.8)		

*TOTAL (n=144)*	*87.5 *	*7 *	*18.1 *	*0.2 *	*4.8 (3.4-6.9)*	*31.8 (16.6-60.9)∗*
	*(81.1-91.9)*	*(4.3-11.2)*	*(12.6-25.1)*	*(0.1-0.3)*		

## Data Availability

The data used to support the findings of this study are included within Table 2 and the supplementary information file (available here), “Supplementary Materials”, Figure 3 data.

## Supplementary Materials

Supplementary Materials Data used for the construction of Figure 3. The acupoints that match PCV (YES) and those that do not match (NO) have been counted.
