# Supplementary material for: Acupuncture Points and Perforating Cutaneous Vessels Identified Using Infrared Thermography: A Cross-Sectional Pilot Study
Source: Evid Based Complement Alternat Med. 2019 Mar 21;2019:7126439. doi: 10.1155/2019/7126439 (PMC6448333; doi:10.1155/2019/7126439)
Supplement: Supplementary Materials — Data used for the construction of Figure 3. The acupoints that match PCV (YES) and those that do not match (NO) have been counted. [file 7126439.f1.docx]

|  | BL37 | BL40 | KI10 | ST31 | ST32 | ST33 | ST34 | SP10 | SP11 | GB31 | LR9 | LR10 |  |  |  |
| --- | --- | --- | --- | --- | --- | --- | --- | --- | --- | --- | --- | --- | --- | --- | --- |
| Sample number |  |  |  |  |  |  |  |  |  |  |  |  |  |  |  |
| 1 Right | 1 | 1 | 1 | 1 | 1 | 0 | 1 | 1 | 1 | 1 | 1 | 1 |  | 126 =YES | 144 POINTS |
| 1 Left | 1 | 1 | 1 | 1 | 1 | 1 | 1 | 1 | 1 | 1 | 1 | 1 |  | 18 = NO |  |
| 2 Right | 1 | 1 | 1 | 1 | 1 | 0 | 0 | 1 | 1 | 1 | 1 | 1 |  |  |  |
| 2 Left | 0 | 1 | 1 | 1 | 1 | 0 | 0 | 1 | 0 | 1 | 1 | 1 |  |  |  |
| 3 Right | 1 | 1 | 1 | 1 | 1 | 0 | 0 | 0 | 1 | 1 | 1 | 1 |  |  |  |
| 3 Left | 1 | 1 | 1 | 1 | 1 | 1 | 0 | 1 | 1 | 0 | 1 | 1 |  |  |  |
| 4 Right | 1 | 1 | 1 | 1 | 1 | 1 | 0 | 1 | 1 | 1 | 1 | 1 |  |  |  |
| 4 Left | 1 | 1 | 1 | 1 | 1 | 1 | 0 | 1 | 1 | 1 | 1 | 1 |  |  |  |
| 5 Right | 1 | 1 | 1 | 1 | 1 | 0 | 1 | 1 | 1 | 1 | 1 | 1 |  |  |  |
| 5 Left | 1 | 1 | 1 | 1 | 1 | 0 | 1 | 1 | 1 | 1 | 1 | 1 |  |  |  |
| 6 Right | 1 | 1 | 1 | 1 | 1 | 0 | 1 | 1 | 1 | 1 | 1 | 1 |  |  |  |
| 6 Left | 1 | 1 | 1 | 1 | 1 | 1 | 1 | 0 | 1 | 1 | 1 | 1 |  |  |  |
| Control number |  |  |  |  |  |  |  |  |  |  |  |  |  |  |  |
| 1 Right | 1 | 0 | 0 | 0 | 0 | 0 | 0 | 1 | 0 | 0 | 0 | 0 |  | 26=YES | 144 POINTS |
| 1 Left | 0 | 0 | 0 | 0 | 0 | 0 | 0 | 1 | 0 | 0 | 0 | 0 |  | 118=NO |  |
| 2 Right | 1 | 0 | 0 | 0 | 0 | 0 | 0 | 1 | 0 | 1 | 0 | 0 |  |  |  |
| 2 Left | 0 | 0 | 0 | 0 | 0 | 0 | 0 | 0 | 0 | 0 | 0 | 0 |  |  |  |
| 3 Right | 1 | 0 | 0 | 0 | 0 | 1 | 0 | 0 | 0 | 0 | 0 | 1 |  |  |  |
| 3 Left | 0 | 0 | 0 | 0 | 0 | 1 | 0 | 0 | 0 | 0 | 1 | 0 |  |  |  |
| 4 Right | 0 | 1 | 0 | 0 | 1 | 0 | 0 | 1 | 0 | 0 | 0 | 0 |  |  |  |
| 4 Left | 0 | 0 | 0 | 0 | 0 | 0 | 0 | 0 | 0 | 1 | 0 | 0 |  |  |  |
| 5 Right | 1 | 0 | 1 | 0 | 0 | 0 | 1 | 0 | 0 | 1 | 0 | 0 |  |  |  |
| 5 Left | 0 | 0 | 0 | 0 | 0 | 0 | 0 | 0 | 1 | 0 | 0 | 0 |  |  |  |
| 6 Right | 0 | 0 | 0 | 0 | 0 | 0 | 0 | 0 | 1 | 0 | 0 | 1 |  |  |  |
| 6 Left | 0 | 0 | 0 | 1 | 0 | 0 | 0 | 0 | 1 | 1 | 1 | 0 |  |  |  |
|  |  |  |  |  |  |  |  |  |  |  |  |  |  |  |  |
| Presence of perforating cutaneous vessels (PCV) | | | | | 1= YES | | 0= NO |  |  |  |  |  |  |  |  |
